# TRPM7, Magnesium, and Signaling

**DOI:** 10.3390/ijms20081877

**Published:** 2019-04-16

**Authors:** Zhi-Guo Zou, Francisco J. Rios, Augusto C. Montezano, Rhian M. Touyz

**Affiliations:** Institute of Cardiovascular and Medical Sciences, BHF Glasgow Cardiovascular Centre, University of Glasgow, Glasgow G12 8TA, UK; z.zou.1@research.gla.ac.uk (Z.-G.Z.); francisco.rios@glasgow.ac.uk (F.J.R.); augusto.montezano@glasgow.ac.uk (A.C.M.)

**Keywords:** TRPM7, magnesium transporters, receptor tyrosine kinases, VEGFR, EGFR

## Abstract

The transient receptor potential melastatin-subfamily member 7 (TRPM7) is a ubiquitously expressed chanzyme that possesses an ion channel permeable to the divalent cations Mg^2+^, Ca^2+^, and Zn^2+^, and an α-kinase that phosphorylates downstream substrates. TRPM7 and its homologue TRPM6 have been implicated in a variety of cellular functions and is critically associated with intracellular signaling, including receptor tyrosine kinase (RTK)-mediated pathways. Emerging evidence indicates that growth factors, such as EGF and VEGF, signal through their RTKs, which regulate activity of TRPM6 and TRPM7. TRPM6 is primarily an epithelial-associated channel, while TRPM7 is more ubiquitous. In this review we focus on TRPM7 and its association with growth factors, RTKs, and downstream kinase signaling. We also highlight how interplay between TRPM7, Mg^2+^ and signaling kinases influences cell function in physiological and pathological conditions, such as cancer and preeclampsia.

## 1. Introduction 

**Figure 1:**
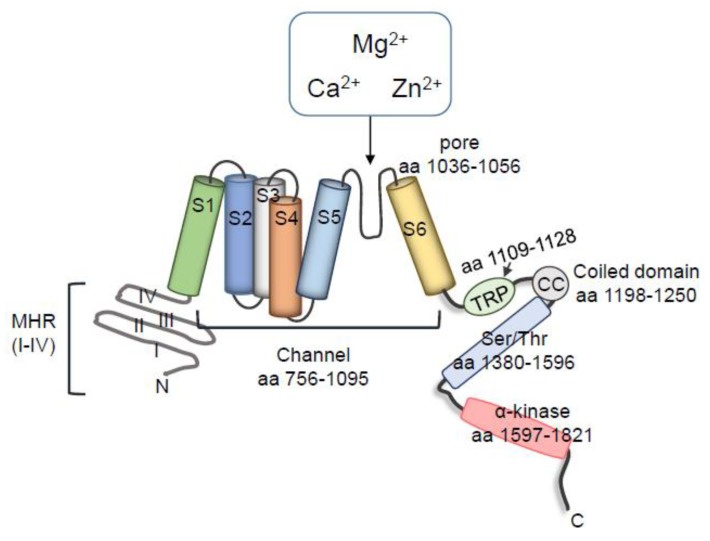
Schematic structure of the TRPM7 channel and kinase. The general structure of the TRPM7 has four Melastatin Homologous Regions (MHR) in the N-terminal domain, and six transmembrane segments (aa 756–1095). The pore (aa 1039–1056) is located between the S5 and S6. The C terminal domain contains the transient receptor potential (TRP) region (aa 1109–1128), common to TRP family members, followed by the coiled coil (CC) domain connecting loop (aa 1198–1250); Serine- and threonine-rich domains (aa 1380–1596), and the α-kinase domain (aa 1597–1821).

The transient receptor potential melastatin 7 (TRPM7) and its close homologue TRPM6 share the unique feature of a cation channel fused to a C-terminal α-kinase domain [1,2,3]. TRPM6 and TRPM7 are present in a tetrameric form and each subunit comprises six transmembrane segments (S1-6), and a channel pore permeable to Mg^2+^, Ca^2+^, and Zn^2+^, located between segments 5 and 6 (Figure 1) [4,5]. TRPM7 is ubiquitously expressed, whereas TRPM6 is mostly expressed in cells from kidneys and intestines. In spite of their similarities, they are not compensated by each other, indicating that they are not redundant. TRPM7 was initially proposed to regulate, and itself to be regulated by, intracellular Mg^2+^ levels, with Mg-ATP below 1mM strongly activating the channel [6]. The importance of TRPM7 in cellular Mg^2+^ homeostasis has been investigated in several cell types, including leukocytes, platelets, vascular smooth muscle cells (VSMCs), cardiomyocytes, cardiac fibroblasts, osteoblasts, and tumor cells, in both physiological and pathological conditions [6,7,8,9,10,11,12,13]. Experiments using TRPM7 overexpressing cells demonstrated important mechanisms underlying the role of TRPM7 in Mg^2+^ homeostasis, and this was confirmed in the recently described crystal structure of mouse TRPM7, where partially hydrated Mg^2+^ ions occupy the center of the conduction pore [3]. However, the importance of TRPM7 in Mg^2+^ homeostasis has also been questioned by some studies, since the deletion of TRPM7 in T cells did not affect acute uptake or the maintenance of total cellular Mg^2+^ [14]. However, this may relate to the cell type studied, because Mg^2+^ is typically regulated by MagT1 in immune cells. 

TRPM6 and TRPM7 have been linked to many signaling pathways. Emerging evidence indicates an important role for these systems in growth factor signaling through receptor tyrosine kinases (RTK). The C-terminal α-kinase domain induces phosphorylation of many downstream targets involved in RTK signaling including annexin A1, calpain II, myosin II, PLCγ2, Src, and SMAD2 [15], and regulates epigenetic modifications [16]. TRPM7 kinase domain may also influence TRPM7 channel function, although the exact mechanisms remain unclear [17,18,19]. 

In addition to controlling Mg^2+^ transport across cell membranes, TRPM7, together with other Ca^2+^ channels, regulates Ca^2+^ influx in many cell types [20,21]. In angiotensin II (Ang II)-stimulated cardiac fibroblasts, TRPM7 is functionally active and controls both Mg^2+^ and Ca^2+^ influx [9]. In neuroblastoma cells, bradykinin-induced activation of TRPM7 mediates Ca^2+^ influx in a kinase-independent manner [22] and Ca^2+^ entry in macrophages triggered by lipopolysaccharide (LPS) is controlled by TRPM7, as observed in a cell-specific deletion model using a Cre/Lox system [23]. The kinase domain of TRPM7 also plays an important role in Ca^2+^ homeostasis by modulating store-operated calcium channels (SOCE) [24,25]. 

TRPM7 is also permeable to Zn^2+^ and may be an important regulator of cellular Zn^2+^ dynamics. This was originally identified in mouse cortical neurons, where activation of TRPM7 channels increased intracellular Zn^2+^, and TRPM7 knockdown using a short hairpin RNA reduced TRPM7-like current and intracellular Zn^2+^ concentration [26]. Additionally, a recent study showed that TRPM7 is an intracellular Zn^2+^ storage vesicle, which sequesters Zn^2+^ during cytosolic overload, and releases Zn^2+^ under oxidizing conditions [27]. Interestingly, other channels from the TRPM family are also permeable to Zn^2+^, such as TRPM2, TRPM3, and TRPM5 [28,29,30].

Receptor tyrosine kinases (RTKs), through which growth factors signal, are membrane-associated receptors [31]. Upon growth factor binding, autophosphorylation of RTKs induces phosphorylation and activation of intracellular non-receptor tyrosine kinases that influence phosphorylation of downstream target proteins. The activation of RTKs is involved in critical signaling pathways and cell functions, including proliferation, differentiation, migration and contraction, processes also dependent on Mg^2+^ and Ca^2+^. The RTK family comprises major growth factor receptors, such as epidermal growth factor receptor (EGFR), vascular endothelial growth factor receptor (VEGFR), platelet-derived growth factor receptor (PDGFR), nerve growth factor receptor (NGFR), fibroblast growth factor receptor (FGFR), and insulin-like growth factor receptor (IGFR) [32].

In 2009, Bindels and colleagues demonstrated that EGF activates EGFR signaling and promotes TRPM6 translocation from the cytosol to the membrane in kidney cells, through Src family tyrosine kinases [33]. These effects were abolished by the monoclonal antibody specific to EGFR (cetuximab) [33,34]. Similar effects were observed in mice treated with erlotinib [35] and in mammary epithelial cells treated with tyrphostin AG1478, inhibitors of EGFR tyrosine kinase [36]. These studies were amongst the first to show a relationship between TRPM6, Mg^2+^ and growth factors and might explain the severe hypomagnesemia observed as a side effect in cancer patients treated with EGFR inhibitors [37]. Most clinical trials reported electrolyte disorders, particularly hypomagnesemia and hypocalcemia, in cancer patients treated with EGFR inhibitors [34]. Whether EGFR inhibitors also influence TRPM7 in cancer patients is unclear. 

## 2. Transient Receptor Potential Melastatin 7 Cation Channel (TRPM7)

### 2.1. Characteristics and Regulation of TRPM7

Among the more than 300 mammalian ion channels, only TRPM7 and TRPM6 have a C-terminal kinase, which belongs to a specific subfamily of atypical protein kinases (APKs), known as α-kinases, displaying little amino acid sequence similarity to conventional protein kinases [3,38,39]. To date very few α-kinases have been identified. Those that have been described include eukaryotic Elongation Factor 2 Kinase (eEF2K), alpha-kinase 1 (lymphocyte alpha-kinase, LAK or ALPK1), alpha-kinase 2 (heart alpha-kinase, HAK or ALPK2) and alpha-kinase 3 (muscle alpha-kinase, MAK or ALPK3), TRPM6 and TRPM7 [38]. The crystal structure of TRPM7 shows that the N-terminal central catalytic core is structurally similar to the classical protein kinase, whereas the structure of the C-terminal lobe resembles metabolic enzymes with ATP-grasp folds [3,39]. The TRPM7 α-kinase predominantly phosphorylates serine/threonine residues on α-helices. Since the cytoplasmic domain is rich in serine/threonine residues, activation of TRPM7 can induce autophosphorylation as well as phosphorylation of downstream targets (Figure 1) [40,41]. TRPM7-kinase phosphorylates annexin-A1 (Ser5) [42]; myosin IIA (Thr1800, Ser1803 and Ser1808) [43]; eukaryotic elongation factor 2 cognate kinase (eEF2-K) (Ser77) under low Mg^2+^ concentrations [44]; SMAD2 (Ser465/467) [15]; and phospholipase Cγ2 (PLCγ2) (Ser1164) [45]. TRPM7 is cleaved by caspases at Asp1510, dissociating the kinase from the conducting pore, with the cleaved channel exhibiting enhanced activity, and the kinase domain retaining phosphotransferase activity [46]. TRPM7 cleaved fragments translocate and accumulate in the nucleus, where they phosphorylate specific serines and threonines on histones, affecting gene expression. It seems that different cleaved fragments are expressed in different tissues, implying a distinct role for TRPM7 cleaved fragments in a tissue-specific manner [16]. A similar phenomenon has been described for TRPM6 kinase [47]. 

### 2.2. Interactions between TRPM7 Kinase and TRPM7 Channel Domains

**Figure 2:**
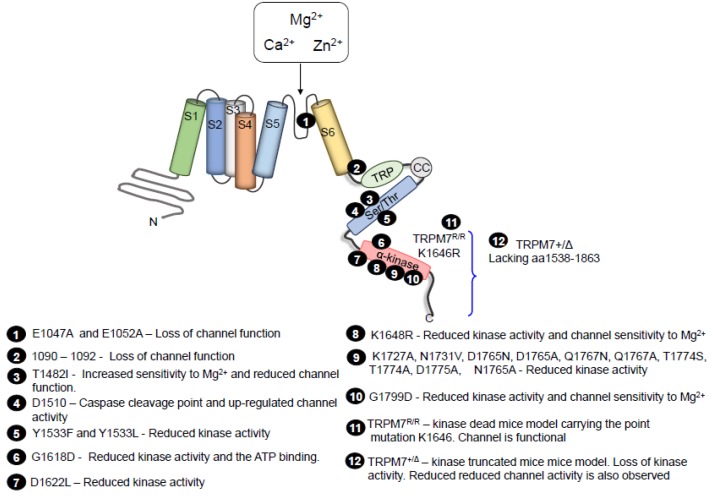
Schematic overview of TRPM7 mutations. Mutations that have been identified are highlighted. Numbers indicate the single amino acids or regions that affect TRPM7 channel or kinase activity [17,19,46,48,49,54,55,56,57,58,59].

Despite intense investigations, there are still controversies regarding the functional significance of the coupling between the α-kinase and the channel domain of TRPM7. Mutations at specific sites that disrupt kinase activity do not affect TRPM7 channel activity and sensitivity to inhibition by divalent cations (Figure 2). In addition, there were no significant differences in channel activity and Ca^2+^ influx between wild type and TRPM7 kinase mutant cells [48]. These data were confirmed in in vivo experiments, where TRPM7 kinase-dead mutant mice have normal serum Mg^2+^ levels and normal development [15,19,48]. However, many studies support a link between TRPM7 channel and its C-terminal kinase. In HEK293 cells overexpressing mutant human TRPM7 K1648R and G1799D, activity of the kinase domain and function of the channel domain are altered by changes in sensitivity to intracellular Mg^2+^ [49]. Interplay between the kinase and channel is supported by studies showing that: (i) cleavage of TRPM7 kinase is associated with increased cation channel activity [46], (ii) Mg^2+^ nucleotide modulation of TRPM7 channel through the kinase domain [50,51], and (iii) regulation of TRPM7 by cAMP/PKA, which requires a functional kinase domain [52]. Additionally, an in vivo model carrying a truncated kinase domain showed reduced intracellular Mg^2+^, hypomagnesemia [17] and increased sensitivity to Ca^2+^ in mast cells [53]. Therefore, it seems that the catalytic activity of the kinase is not essential for channel gating, but somehow it modulates and “fine tunes” channel activity and sensitivity to Mg^2+^ nucleotides. 

### 2.3. Relationship between TRPM6 and TRPM7

**Figure 3:**
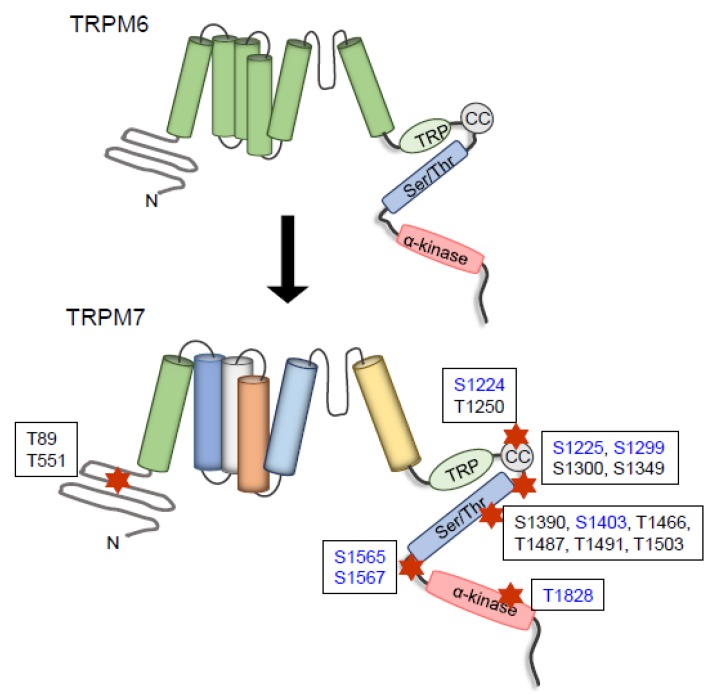
Transphosphorylation of TRPM7 by TRPM6. TRPM6 is able to induce phosphorylation of TRPM7 in the indicated residues. Red stars indicate the location of the residues. Residues in blue are also residues of autophosphorylation.

In the plasma membrane, TRPM7 functions as a homodimer, but in some conditions it also heterodimerizes with TRPM6. In some cell-based systems, TRPM6 seems to require TRPM7 for full activation and in the complexed state has been termed TRPM6/TRPM7 [60]. The crucial role of TRPM7 in the complex formation was demonstrated in molecular studies, where TRPM6 deficiency in trophoblast stem cells results in a reduction in TRPM6/7 currents, while deficiency in TRPM7 completely abolished the TRPM6/7 currents [53]. TRPM6 and TRPM7 possess several phosphorylation sites and can be phosphorylated independently of autophosphorylation, TRPM7 can also be transphosphorylated by TRPM6 (Figure 3) [61]. This cross activation was demonstrated in co-transfection experiments, which showed that TRPM6 expression was able to transphosphorylate serine and threonine motifs in TRPM7-K1646R kinase dead mutant. Transphosphorylation of TRPM6 by TRPM7 is very weak. These phosphorylated residues are present in the N-terminal, channel, and C terminus, including the kinase domain of the TRPM7-K1646R [61]. These phenomena have been demonstrated in cell models but the (patho) physiological significance in vivo remains to be determined. TRPM7 seems to interact with other Mg^2+^ channels. In DT40 B cells and colon carcinoma cells, TRPM7 deficiency was associated with increased expression of MagT1 [62,63,64]. 

### 2.4. TRPM7 and Cell Function 

The indispensable role of TRPM7 in cellular biology is likely linked to its important role in the regulation of homeostasis of divalent cations Mg^2+^, Zn^2+^, and Ca^2+^. Mg^2+^ and Zn^2+^ are catalytic and structural cofactors of numerous enzymes and are major regulators of signaling molecules, DNA stability, cell cycle and transcription factors [65,66]. Deficiency of Mg^2+^ and Zn^2+^ suppress cell cycle progression leading to growth failure [67,68]. Mg^2+^ supplementation and overexpression of Mg^2+^ transporters rescued growth impairment caused by TRPM7 deficiency [17,62]. Ca^2+^, which is also influenced by TRPM7, is critically involved in controlling cell function including cell proliferation, contraction, secretion, migration and differentiation [69,70,71]. Associated with many of the Ca^2+^- and Mg^2+^- regulated signaling pathways is the activation of tyrosine kinases [35,72,73,74,75]. In vascular cells, TRPM7 appears to be the most important cation channel involved in controlling [Mg^2+^]_i_. TRPM7 is regulated by vasoactive factors, such as AngII, aldosterone, endothelin-1 and bradykinin [76,77,78,79,80]. Ang II regulates vascular TRPM7 acutely by inducing phosphorylation and chronically by increasing expression at the mRNA and protein levels [76,79]. Also, siRNA downregulation of vascular TRPM7 caused a reduction in [Mg^2+^]_i_ and attenuated Ang II-mediated VSMC growth [7]. VSMCs from hypertensive rats exhibit reduced TRPM7 expression and decreased TRPM7 activation, as assessed by translocation of annexin-1 (TRPM7 kinase target) to the membrane [79]. Findings from cell-based studies and animal models indicate that TRPM7 activation and increased Mg^2+^ influx are vasoprotective as they protect against vascular calcification, oxidative stress and fibrosis [81]. 

## 3. Receptor Tyrosine Kinase Signaling and Mg^2+^

### 3.1. Characteristics and Regulation of Receptor Tyrosine Kinases 

Protein kinases induce phosphorylation by catalyzing the transfer of phosphate from adenosine triphosphate (ATP) to serine, threonine and tyrosine residues on protein substrates and are key enzymes in the regulation of intracellular signaling pathways [82,83]. These ATP- and phosphate-dependent processes have an obligatory requirement for Mg^2+^. Data from the human genome revealed that of the proteins phosphorylated on tyrosine residues, ≈58 are receptor tyrosine kinases and ≈32 are non-receptor tyrosine kinases [32,84]. All RTKs have a similar molecular architecture, comprising three major domains, including an extracellular ligand-binding domain, an intracellular tyrosine kinase domain and a transmembrane domain [31]. RTKs are typically activated by growth factors [85,86,87,88].

**Figure 4:**
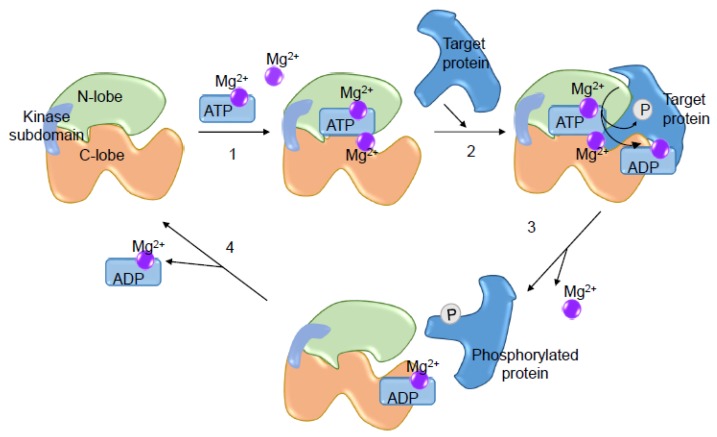
Importance of Mg^2+^ for the kinase catalytic activity. Two Mg^2+^ molecules are required for enzymatic activity and phosphoryl transfer: one bound to ATP (Mg-ATP) situated between the small (N-lobe) and large lobe (C-Lobe) of the kinase domain, and another Mg^2+^ situated in the catalytic loop. (**1**) Mg-ATP is the first to bind to the enzyme followed by Mg^2+^; (**2**) the kinase binds to the protein substrate and catalyzes the transfer the phosphoryl group; (**3**) phosphorylated protein and Mg^2+^ are released; and (**4**) Mg-ADP is released and the catalytic cycle is finalized.

Magnesium is a crucial divalent cation required for the activity of protein kinases, including RTKs (VEGFR, EGFR, FGFR, PDGFR) and non-receptor tyrosine kinases (Src, Abl, Jak, FAK, SOCS) [89]. In cell-based studies, high Mg^2+^ concentration causes increased tyrosine kinase activity. Data obtained with crystallography experiments revealed that two Mg^2+^ molecules are required for enzyme activity and phosphoryl transfer: one bound to ATP (Mg-ATP) situated between the small and large lobes of the kinase domain, bound to β and γ-phosphates and to the aspartate of the DFG (Asp-Phe-Gly), which are the first residues to be activated in protein kinases; and in high [Mg^2+^]_i_ conditions, another Mg^2+^ binds to α and γ-phosphates and to the asparagine amide nitrogen within the catalytic loop [90]. The importance of Mg^2+^ in the regulation of kinase activity occurs in four steps: (i) Mg-ATP binds to the enzyme; (ii) the kinase binds to the protein substrate and catalyzes the transfer of the phosphoryl group; (iii) phosphorylated protein and Mg^2+^ are released; and (iv) Mg^2+^-ADP is released and the catalytic cycle is completed [91] (Figure 4). 

### 3.2. Growth Factors and Receptor Tyrosine Kinase Signaling

**Figure 5:**
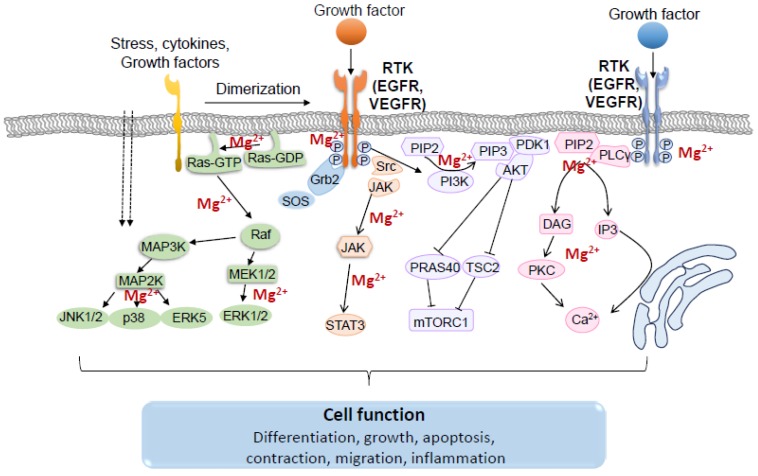
Schematic representation of main signaling pathways activated by growth factor signaling through receptor tyrosine kinases (RTK) and the importance of Mg^2+^. Ligand-induced dimerization triggers transphosphorylation of tyrosine residues located in the receptor chain, resulting in activation of RTKs. Intracellular signaling cascades activated by RTKs include the MAPK pathway, the PI3K/AKT/mTOR pathway, the JAK/STAT pathway, the PLCγ/PKC pathway and the Src pathway. The functional response of these signals plays an essential role in the regulation of many physiological processes. Dotted arrows indicate activation through the cell membrane; Solid arrows indicate activation of target protein; T indicates inhibition.

Following binding of growth factors to their specific RTKs, cytoplasmic proteins containing Src homology region 2 (SH2) or phosphotyrosine-binding (PTB) domains are recruited to the cell membrane. These recruited proteins either have intrinsic enzymatic activity, such as Src and PLCγ, or serve as docking proteins that function as “assembly platforms” and recruit additional enzymes [32,75,85,92]. Activated RTKs are able to trigger a wide range of downstream signaling pathways, including RAS/RAF/MEK/MAPK, PLCγ/PKC, PI3K/AKT/mTOR, and JAK/STAT (Figure 5) [93].

Activated RTKs bind to the adaptive protein growth factor receptor-bound 2(Grb2) and recruit Son of Sevenless homolog protein (SOS), which interacts with its downstream target Ras, a small GTP binding protein, and transforms it to the active conformation by exchanging GDP for GTP [94]. Ras recruits the serine/threonine protein kinase, Raf, to the membrane, where it becomes activated by phosphorylation. Once activated, Raf, a MAP3K, phosphorylates MAP2Ks, MEK1 and MEK2, at specific serine residues, and activated MEK1/2 in turn catalyzes phosphorylation of ERK1/2 at threonine and tyrosine residues [95]. Activated ERK1/2 phosphorylates many downstream target proteins and transcription factors involved in cell function [32,96]. 

The phosphoinositol-3-kinase (PI3K)/Akt/mammalian target of rapamycin (mTOR) signaling pathway is also initiated by binding of growth factors to RTKs. Activated RTKs recruit the PI3K to the plasma membrane where the PI3K subunit p110 catalyzes phosphorylation of phosphatidylinositol 4,5-bisphosphate (PIP2) to phosphatidylinositol 3,4,5-triphosphate (PIP3). PIP3 then provides docking sites for signaling proteins, including AKT and 3-phosphoinositide-dependent kinase 1 (PDK1) [97,98]. Activated AKT phosphorylates many other downstream proteins, such as glycogen synthase kinase 3 (GSK3), the forkhead family of transcription factors (FOXOs) and mTOR. Multiple components of the PI3K/AKT/mTOR pathway activated by RKTs play a pivotal role in the regulation of cell growth, proliferation and differentiation [93,98,99,100].

Another signaling system linked to RTKs is PLCγ/protein kinase C (PKC). Upon growth factor stimulation, the phosphorylated tyrosine residues of RTKs interact with SH2 domains of PLCγ and lead to its activation. PLCγ then hydrolyses PIP2 into two second messengers, inositol 1,4,5-trisphosphate (IP3) and diacylglycerol (DAG). IP3 binds to its receptor on the endoplasmic reticulum (ER) surface, whereas DAG mediates activation of PKC [101,102]. This process has a key role in regulating intracellular Ca^2+^ through multiple Ca^2+^ channels [103,104,105]. PLCγ/PKC is also involved in the regulation of cell proliferation, contraction and migration [106,107,108].

The Janus kinase/signal transducer and activator of transcription (JAK/STAT) and the Src family kinases (SFKs) are additional signaling pathways associated with RTK activation. Src family members, including Src, Fyn and Yes, are recruited on RTKs, such as EGFR, FGFR and IGFR, and transmit mitogenic signals to regulate DNA synthesis, cell survival, cell adhesion, motility and growth [32]. Once activated, STATs enter the nucleus and bind to specific regulatory sequences in target genes regulating transcription [109]. 

## 4. Cross-Talk between TRPM6/7, Receptor Tyrosine Kinases and Signaling Kinases

### 4.1. Regulation of TRPM6/7 by Receptor Tyrosine Kinases 

Upon ligand binding, VEGFR and EGFR undergo receptor dimerization and tyrosine phosphorylation leading to recruitment of effector proteins and activation of downstream cascades. These processes are highly regulated and are dependent on intracellular Mg^2+^. Increased RTK activation and abnormal growth factor-mediated tyrosine kinase signaling is associated with uncontrolled cell proliferation in cancer [110]. Accordingly, inhibitors of VEGFR and EGFR tyrosine kinases have been used to treat several cancers. However unexpectedly these drugs have been associated with unwanted secondary effects, including hypertension, cardiovascular and renal toxicity, and electrolyte disorders [110,111,112,113].

One of the most significant electrolyte abnormalities associated with EGFR inhibitors in the treatment of cancer is renal magnesium wasting and hypomagnesemia. In extreme cases, patients need intravenous magnesium treatment and withdrawal of the EGFR inhibitor. This has major impact on the effectiveness of cancer treatment [110,111,112,113,114]. Molecular mechanisms underlying these side effects have been attributed to abnormal function of TRPM6, phenomena that were first described in patients carrying a mutation in the EGF gene and who have severe hypomagnesemia and cognitive disability. EGF binding to EGFR promotes TRPM6 translocation from cytosol to membrane in kidney cells [33]. This process was blocked by the EGFR monoclonal antibody, cetuximab [33,34]. Hence, EGF-EGFR signaling involves TRPM6, which promotes Mg^2+^ transport into cells. Inhibition of these processes with EGFR inhibitors decreases TRPM6 activity and reduces Mg^2+^ influx into cells, promoting Mg^2+^ excretion in the kidney with resultant Mg^2+^ wasting and hypomagnesemia. 

Emerging evidence suggests that TRPM7 may also be regulated by growth factor signaling through EGFR, as well as VEGFR [115,116,117]. Studies in transfected chinese hamster ovary (CHO) cells showed inhibition of TRPM7 channel activity by EGF through mechanisms involving PLCγ and PIP2 [117]. However, Gao et al. found that in pulmonary cancer cell lines, EGF, through its receptor, enhanced the cell membrane protein expression and currents of TRPM7, a process associated with cell migration [118]. The difference in models and cell lines may explain the conflicting data obtained in these two studies. In hippocampal neurons, nerve growth factor (NGF) signals through receptor tyrosine kinase TrkA, reducing the outward rectifying TRPM7-like current, effects blocked by inhibitors of TrkA and PLC [119]. TrkA activation by NGF prevented upregulation of TRPM7 expression through PI3K in hippocampal neurons subjected to ischemia-reperfusion and oxygen-glucose deprivation [120]. Moreover, PDGF stimulation was shown to increase TRPM7 expression in HSC-T6 hepatic stellate cells. The non-specific TRPM7 inhibitor 2-aminoethyl diphenyl borinate (2-APB) diminished PDGF-mediated activation of p-AKT and p-ERK, further suggesting a regulatory role for TRPM7 upstream of AKT and ERK [121]. 

**Figure 6:**
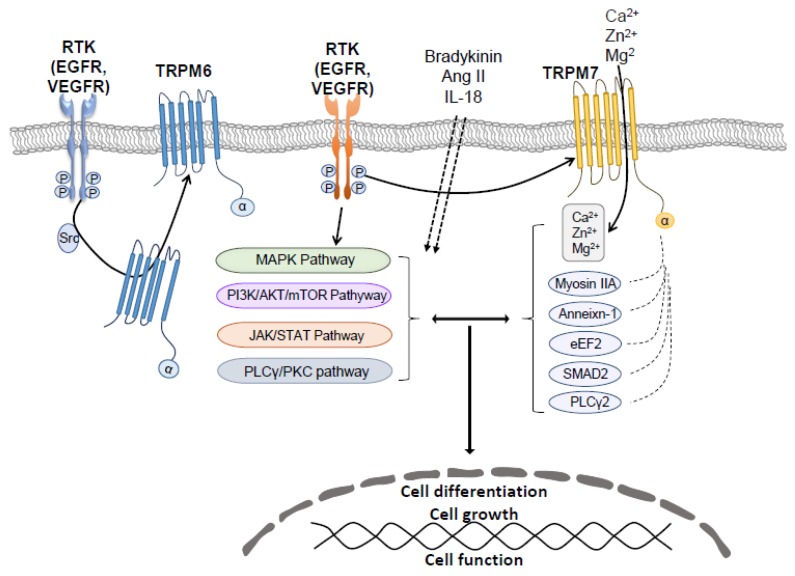
Cross-talk between Receptor Tyrosine Kinases (RTK) downstream signaling and TRPM7. TRPM7 has the dual properties of acting as an ion channel mainly permeable to Zn^2+^, Mg^2+^ and Ca^2+^, and as a cytoplasmic kinase, which phosphorylates annexin-1, myosin IIA heavy chain, eEF2, SMAD2, and PLCγ2. Through either its channel or kinase, TRPM7 participates in RTK downstream signaling pathways. On the other hand, ligand induced activation of RTKs, such as EGFR, regulates the activity of TRPM7 or TRPM6. Signaling by bradykinin, Ang II, and IL-18 also influence TRPM7 activity. Dotted arrows indicate activation through the cell membrane; Solid arrows indicate activation of target protein; T indicate inhibition.

RTKs induce phosphorylation of downstream kinases that influence TRPM7 activity (Figure 6). However these processes seem to be related to changes in the intracellular concentration of Mg^2+^, because when [Mg^2+^]_i_ is low, PLC activation is associated with TRPM7 inhibition, whereas in normal [Mg^2+^]_i_ conditions, activation of PLC increases TRPM7 currents [122]. In prostate cells, TRPM7 expression was mediated by Ca^2+^-dependent activation of ERK, since ERK inhibition reduced TRPM7 expression [123]. Ang II, which transactivates TKRs, increased TRPM7 expression through the AT1 receptor and ERK [124]. Bradykinin, another vasoactive peptide, regulates TPRM7 and its downstream target annexin-1 through PLC and c-Src dependent pathways, which have an important role in VSMC Mg^2+^ homeostasis and cell migration and invasion [78]. The inflammatory mediator interleukin-18 (IL-18) activated TRPM7 currents and upregulated TRPM7 expression in an ERK1/2-dependent manner, processes that influence osteogenic differentiation of VSMCs [125]. In HEK293 cells, interleukin-6 (IL-6) inhibited TRPM7 currents through JAK2-STAT3 signaling and appeared to be independent of the TPRM7 α-kinase domain. Regulation of TRPM7 by IL-6 signaling may result from JAK2-STAT3 mediated phosphorylation of TRPM7 [126]. 

### 4.2. Regulation of Signaling Kinases by TRPM7 

While kinases regulate TRPM7, these kinases themselves may be targets of TRPM7. MAP kinases are regulated by TRPM7. In human endothelial cells, downregulation of TRPM7 using a siRNA approach was associated with increased ERK1/2 phosphorylation with no effects on p38MAPK or JNK activation [127]. On the other hand, in HEK293 cells overexpressing TRPM7, ERK1/2 phosphorylation was reduced, while phosphorylation of p38MAPK and JNK was increased [128]. In a rat hepatic stellate cell line (HSC-T6), upregulation of TRPM7 promoted PDGF-induced activation of ERK1/2 and AKT pathways [121]. In mouse cortical astrocytes and in rat VSMCs, TRPM7 siRNA was associated with reduced ERK1/2 phosphorylation [129,130]. Additionally, TRPM7 siRNA reduced activation of Src, p38 MAPK, ERK1/2 and JNK in a breast cancer cell line [131,132]. 

A regulatory role of TRPM7 in MAPK signaling has also been observed using pharmacological approaches in cancer tissue and cell lines. In glioblastoma cells, naltriben, a pharmacological activator of TRPM7 channel, enhanced ERK1/2 phosphorylation and proliferation, with no effect on the PI3K/AKT pathway (150). Carvacrol, a non-specific inhibitor of TRPM7, suppressed phosphorylation of ERK1/2 and AKT, reducing cell proliferation [133,134,135]. Thus, there is accumulating evidence supporting a role for TRPM7 in the regulation of MAPK signaling. 

The PI3K/AKT pathway is also affected by TRPM7. In mouse chondrocytes, overexpression of TRPM7 stimulated the AKT pathway, while TRPM7 silencing inhibited AKT signaling [136]. In human osteoblasts, Mg^2+^-induced increase in mRNA expression of chemotaxis-related genes was attenuated by TRPM7 siRNA and PI3K inhibition, suggesting a link between TRPM7 and PI3K activation mediated by Mg^2+^.

STAT3 is also modulated by TRPM7. TRPM7 siRNA reduced activation of STAT3 (Tyr705) by affecting phosphorylation of the upstream protein JAK2, a non-receptor tyrosine kinase [137]. Reduced TRPM7 expression was associated with inhibition of Notch activation (Notch1, JAG1, Hey2, and Survivin), involved in angiogenesis and tumor growth mediated by VEGFR and EGFR [138,139]. In breast cancer cells, silencing TRPM7 reduced EGF-induced STAT3 activation and decreased phosphorylation levels of Src, with reduced TRPM7-mediated migration and invasion [131,132]. 

## 5. TRPM7and Kinase Signaling—Pathophysiological Implications

TRPM7-regulated Mg^2+^ has versatile biological functions, contributing to all vital cellular processes, including stability of tertiary structures of DNA and RNA, energy metabolism, enzyme activity, signaling, cell cycle progression and cell differentiation [140,141]. The significance of TRPM7 in development and cell viability was demonstrated in mice with global deletion of TRPM7. TRPM7 knockout mice are embryonic lethal and cardiac-targeted knockout of TRPM7 causes impaired embryonic development of the heart [14,142]. The regulatory role of TRPM7 in cell differentiation has been highlighted in various cell types. In mesenchymal stromal cells, TRPM7 mediates shear stress and silencing TRPM7 accelerates osteogenic differentiation [143]. TRPM7 was also shown to mediate differentiation in hepatic cells, lung fibroblasts, dental pulp stem cells and T cells [14,144,145,146]. Some of these studies investigated underlying mechanisms of the involvement of TRPM7 in cell differentiation. In addition to the contribution of Ca^2+^ and Mg^2+^, an interaction between TRPM7 and RTK downstream effectors, including PI3K, AKT and ERK1/2, have been implicated [81,125,145,147,148,149]. 

Developmental experiments using zebrafish models of TRPM7 mutants showed abnormal pancreas development, which was associated with increased expression of Suppressor of cytokine signaling (SOCS)-3a, the zebrafish homologue to the human SOCS3, which is part of a classical negative feedback system that influences cytokine signal transduction by reducing activation of JAK-STAT3. Mg^2+^ supplementation partially attenuated the phenotype [150]. Using genetic and pharmacologic inhibitors, 2-APB or TRPM7-siRNA, it was further shown that TRPM7 modulates effects of PDGF-BB on cell proliferation by regulating activity of cell cycle proteins, including cyclin D1, PCNA and CDK4, through mechanisms that involve tyrosine kinases, ERK1/2 and PI3K [121].

The effects of TRPM7 in pathologic cell differentiation has been intensively investigated in cancer cells, including pancreas, ovary, breast and adenocarcinoma of lungs and prostate [118]. Expression of TRPM7 in these cancers was associated with increased expression of proliferative markers. Epithelial-mesenchymal transition is involved in fibrosis and cancer metastasis and involves EGF activation, effects attenuated by TRPM7 siRNA by mechanisms involving ERK1/2 and STAT3 [132]. 

Cancer stem cells are cells found within tumors and hematological cancers that have features of normal stem cells, particularly the ability to self-renew and differentiate into multiple cell types [151]. They are rare immortal cells within tumors and are able to induce growth of new tumors and metastases. They are found in numerous tumors and have been considered attractive targets for anti-cancer therapy [152]. Many pathways have been implicated in the regulation of stem cell self-renewal and oncogenesis, including signaling through Notch, Wnt and sonic hedgehog (Shh) [151,152]. In epithelial tumors, epithelial-mesenchymal transition (EMT) seems to be important in cancer stem cell function. Recent evidence indicates that cancer stem cell regulation involves TRPM7-dependent pathways. In lung cancer cells, TRPM7 expression was increased and associated with enhanced SOX2, KLF4, and CD133, Hsp90α, uPA, and MMP2 [153]. TRPM7-silencing inhibited epithelial-to-mesenchymal transition (EMT), suppressed stemness markers and phenotypes, and attenuated activation of the Hsp90α/uPA/MMP2 axis. These effects were ameliorated by waixenicin A, a bioactive extract of soft coral, which downregulated TRPM7 and oncogenic markers. Hence, waixenicin A was suggested as an anticancer therapy, by inhibiting lung cancer stem cells through TRPM7 inhibition [153]. In glioma tumorigenesis cancer stem cells, TRPM7 activates JAK2/STAT3- aldehyde dehydrogenase1 (ALDH1) and Notch signaling pathways, contributing to the regulation of cell proliferation, migration and invasion [137]. TRPM7 has also been implicated in epithelial-mesenchymal transition ovarian cancer stem cells, through calcium-related PI3K/AKT oncogenic signaling [154]. In mesenchymal stromal cells (MSCs), TRPM7 senses mechanical stimuli, such as intermittent fluid shear stress and membrane tension, and regulates Ca^2+^ influx and phosphorylation of signaling kinases, Smad1/5 and p38 MAPK, whereas TRPM7 knockdown decreases MSC proliferation and viability, and induces cell death [143]. Additionally, Mg^2+^ supplementation promoted osteogenic differentiation through the activation of Notch signaling in MSCs, which was decreased by 2-APB [143,155,156,157]. In human dental pulp stem cells, silencing TRPM7 inhibited proliferation, migration, and osteogenic differentiation, supporting a role for TRPM7 in the dental pulp repair process [146]. Together, these studies suggest that dysregulation of TRPM7 is important in cancer stem cell regulation and that TRPM7 may be a therapeutic target for oncogenesis [158].

Interactions between RTKs, such as VEGFR and EGFR, TRPM6/7 and Mg^2+^, have also been implicated in preeclampsia. In a study examining placentas from women with preeclampsia compared to placentas from normotensive women, pre-eclamptic placentas had reduced expression of TRPM6 and TRPM7 [159], which was linked to altered cellular Mg^2+^ homeostasis. These phenomena were associated with decreased placental VEGF expression in preeclampsia versus normotensive pregnancies, effects normalized by treatment with MgSO_4_ [160]. Together these findings suggest an important relationship between VEGF, TRPM6/7 and Mg^2+^ in preeclampsia. This is clinically important because it provides a mechanistic basis for the therapeutic use of MgSO4 as the drug of choice for preeclampsia [161].

## 6. Conclusions and Perspectives 

TRPM7 and TRPM6 are unique proteins characterized by their channel and kinase domains, which differentially regulate cell signaling and function. TRPM6 is mostly expressed in epithelial cells of the kidney and gastrointestinal system, whereas TRPM7 is ubiquitously expressed. In the kidney, TRPM6 and cellular Mg^2+^ homeostasis are regulated by EGF signaling through EGFR. Inhibition of EGFR signaling impairs TRPM6 function leading to renal Mg^2+^ wasting and consequent hypomagnesemia in cancer patients treated with EGFR inhibitors [161]. It is still unclear if similar processes influence TRPM7. Experimental studies, mainly in cell models, indicate that VEGFR and EGFR influence TRPM7 activity and Ca^2+^ and Mg^2+^ mobilization. Changes in TRPM7 activity and altered Mg^2+^ homeostasis have a significant effect on tyrosine kinase signaling. Hence, RTKs influence TRPM7, which in turn may influence tyrosine kinase signaling. Most studies have focused on EGFR signaling, but growing evidence indicates that VEGFR and PDGFR also signal through TRPM7. The putative cross-talk between TRPM7 and RTKs has clinical relevance, because inhibitors of RTKs, including VEGFR and EGFR, are increasingly being used to treat cancer and inflammatory diseases. Hence, knowing mechanisms whereby these drugs cause hypomagnesemia is important so that unwanted side effects can be managed in a mechanism-specific manner. The field of TRPM6/7 and RTKs is still immature and further studies are required to unravel the complex interplay between these systems.
